# Probabilistic record linkage of de-identified research datasets with discrepancies using diagnosis codes

**DOI:** 10.1038/sdata.2018.298

**Published:** 2019-01-08

**Authors:** Boris P. Hejblum, Griffin M. Weber, Katherine P. Liao, Nathan P. Palmer, Susanne Churchill, Nancy A. Shadick, Peter Szolovits, Shawn N. Murphy, Isaac S. Kohane, Tianxi Cai

**Affiliations:** 1 Department of Biostatistics, Harvard T.H. Chan School of Public Health, Boston, MA, USA; 2 University Bordeaux, ISPED, Inserm Bordeaux Population Health Research Center, UMR 1219, Inria SISTM, Bordeaux F-33000, France; 3 Department of Biomedical Informatics, Harvard Medical School, Boston, MA, USA; 4 Division of Rheumatology, Immunology, and Allergy, Brigham and Women’s Hospital, Boston, MA, USA; 5 Computer Science and Artificial Intelligence Laboratory (CSAIL), Massachusetts Institute of Technology, Cambridge, MA, USA; 6 Department of Neurology, Massachusetts General Hospital, Boston, MA, USA; 7 Research IS and Computing, Partners HealthCare, Charlestown, MA, USA

**Keywords:** Medical research, Diagnosis

## Abstract

We develop an algorithm for probabilistic linkage of de-identified research datasets at the patient level, when only diagnosis codes with discrepancies and no personal health identifiers such as name or date of birth are available. It relies on Bayesian modelling of binarized diagnosis codes, and provides a posterior probability of matching for each patient pair, while considering all the data at once. Both in our simulation study (using an administrative claims dataset for data generation) and in two real use-cases linking patient electronic health records from a large tertiary care network, our method exhibits good performance and compares favourably to the standard baseline Fellegi-Sunter algorithm. We propose a scalable, fast and efficient open-source implementation in the ludic R package available on CRAN, which also includes the anonymized diagnosis code data from our real use-case. This work suggests it is possible to link de-identified research databases stripped of any personal health identifiers using only diagnosis codes, provided sufficient information is shared between the data sources.

## Introduction

An increasing quantity and variety of health data, including administrative claims data, electronic health records (EHR) data, and data generated from biomedical research studies, are becoming available for discovery research. For a subset of patients who contribute information to multiple datasets, synthesizing information from these multiple datasets may provide a more complete picture of the patients’ health condition and enable more comprehensive population studies. For example, if research database A contains genomic data and a small set of phenotypic data and claims database B contains a complete list of diagnostic codes associated with patients’ medical histories, linking databases A and B would enable researchers to investigate the role of genomics in a wide range of phenotypic conditions. More broadly, analyses of linked biomedical data could potentially lead to better understanding of disease risks and treatment effects^1,2^. Repurposing existing datasets through record linkage can reduce the cost of research through better exploitation of costly data already collected or curated. Concern for patients’ privacy often makes such research datasets available only in de-identified forms, thus preventing linkage by use of a universal identifier or another nearly-unique personal characteristic of each patient. Our goal here is to investigate the use of informative characteristics of the de-identified data themselves that permit probabilistic linkage among different datasets featuring discrepancies (a discrepancy designates a disparity between two data sources concerning the same information).

Existing record linkage methods can be classified into two broad categories: i) deterministic approaches, where a fixed set of rules determine which records are matching and which are not; and ii) probabilistic methods, where discrepancies can be accounted for and a linkage probability is estimated for each record pair. Many factors can contribute to data discrepancy, such as missing information when a patient seeks care at multiple institutions and not all records are stored in one EHR, or administrative recording errors. Deterministic approaches are highly susceptible to these discrepancies, which has pushed the development of probabilistic record linkage methods^3–6^, for instance to link cohort studies with registry data^7,8^. A statistical framework for probabilistic record linkage was originally proposed by Fellegi & Sunter^9^, formalizing Newcombe’s ideas^10^. Since then, several improvements have been proposed^11–18^, relying on EM-based latent-class algorithms. Other approaches use machine learning methods, but they systematically require representative training data for which the true matching status is known, and this “gold standard” is typically not available^19^. Recently, privacy-preserving record linkage (PPRL) methods relying on encryption of identifiable patient data have also been developed^20–22^.

Regardless of the algorithm, most approaches to patient record linkage rely on individually identifiable health information, such as name, date of birth, and social security number, which the Health Insurance Portability and Accountability Act (HIPAA) classifies as protected health information (PHI). In contrast, datasets generated for research purposes are often de-identified, where all PHI has intentionally been removed to protect patient privacy. The objective of our study is to determine whether it is possible to link different de-identified clinical research datasets using informative characteristics of the clinical research data such as patients’ diagnoses (although linking records in two de-identified datasets does not actually identify any individual patients, caution must be taken since it potentially increases the risk that larger patterns unique to a specific individual may help an ill-intentioned adversary correlate these with other identifying information).

To our knowledge, Bennett *et al.*^4^ published the only attempt to date at doing this. However, they assume that there are no discrepancies between the different data sources, and they never address the choice of priors in their modelling (e.g., on the number of matches, or any other priors). In addition, they require a subsample for which true matches are known, in order to tune certain parameters of their model. Finally, the details of their modelling and of their algorithm are not described in their publication, and the software they use is only available through purchase.

We address these limitations in this study, specifically testing whether it is possible to link different de-identified clinical research datasets using only a patient’s diagnosis codes when discrepancies are present. Many types of data other than diagnoses can also exist in de-identified datasets, such as medications and procedures, and these might even be better than diagnoses for record linkage. However, our focus here is just on diagnoses because of how commonly they appear in research datasets, and because of the widespread use of standard vocabularies (e.g., ICD-9 and ICD-10). Furthermore, diagnosis codes have been shown to uniquely identify most patients within a single dataset^23^. We ignore dates because they are considered PHI, and a de-identified dataset would have no more than the year of an event.

In this study, we evaluate the conditions under which diagnoses contain enough concordant information across datasets to enable linkage. This is important because contrary to PHI and other demographics, which are expected to be the same across datasets (assuming there are no recording errors), diagnosis codes often have valid differences. For example, a patient might be receiving ongoing care for diabetes at one hospital and visit a separate urgent care clinic closer to home for a common cold; or, a healthcare facility might use some refined diagnosis codes internally that are not necessarily sent out for billing, and thus will not always be present in the associated claims data. This means it is important to account for and to model such discrepancies, and that in practice our approach will only be successful in specific situations where the overlapping diagnosis information between the datasets is sufficient. Such situations include linking registry data — previously linked with billing data from a health system source using PHI — with further EHR data, or linking EHR data with a claims database. Here we propose a probabilistic record linkage method that only uses diagnosis codes to estimate a posterior probability of matching between records. We rely on a likelihood framework using a Bayesian approach and a generative model to formally estimate the posterior probability of each patient record pair being a match.

## Results

### Numerical study

We first performed simulation studies based on the 2010 ICD9 code data from 3,153 patients in an un-identifiable claims database from a nationwide US health insurance plan. A total of 2,850 unique ICD9 codes were recorded for these patients with an average of 4.7 codes per patient. To create a second dataset we perturbed the original data with correlated multivariate normal noise as follows: $X^{*}=1_{X\cdot N\left(1,\rho \Sigma \right)+(1-X)\cdot N\left(-3,\rho \Sigma \right)}$ where Σ is the empirical correlation matrix of the 2,850 codes, *X* the original binary data, X^*^ the second dataset, *ρ* the level of perturbation (noise) introduced and 1_*x*_ the indicator function that is 1 if *x* > 0 and 0 otherwise. Then a subset of 2,500 patients from the original data was matched against a subset of 1,253 patients from the perturbed data, with only partial overlap (not all patients have a true match). The proportion of patient overlap varied between different simulation settings. Because the datasets are derived from the same source, the true matches were known.

We compare our method to the Fellegi-Sunter approach, estimated via an E-M algorithm from Grannis *et al.*^15^, itself based on Winkler’s method^11^. Such an approach is still widely used and considered as a competitive unsupervised probabilistic approach for record linkage^19^, even though it is usually used for matching records on a small set of PHI and has never been evaluated for matching clinical data based only on a large number of diagnosis codes. The Fellegi-Sunter method does not support 1-1 matching (the constraint that a given patient record should be matched at most once), yielding exceedingly low positive predictive value (PPV) due to a large number of potential matches for each patient. To reduce this number of false matches in the Fellegi-Sunter method, we also implemented a variant where only the largest matching score is considered as a potential match for each patient (in case of ties at the maximum matching score values, both matches were kept). This enforcement of the 1-1 matching improves statistical performance compared to the original Fellegi-Sunter method.

**Figure 1:**
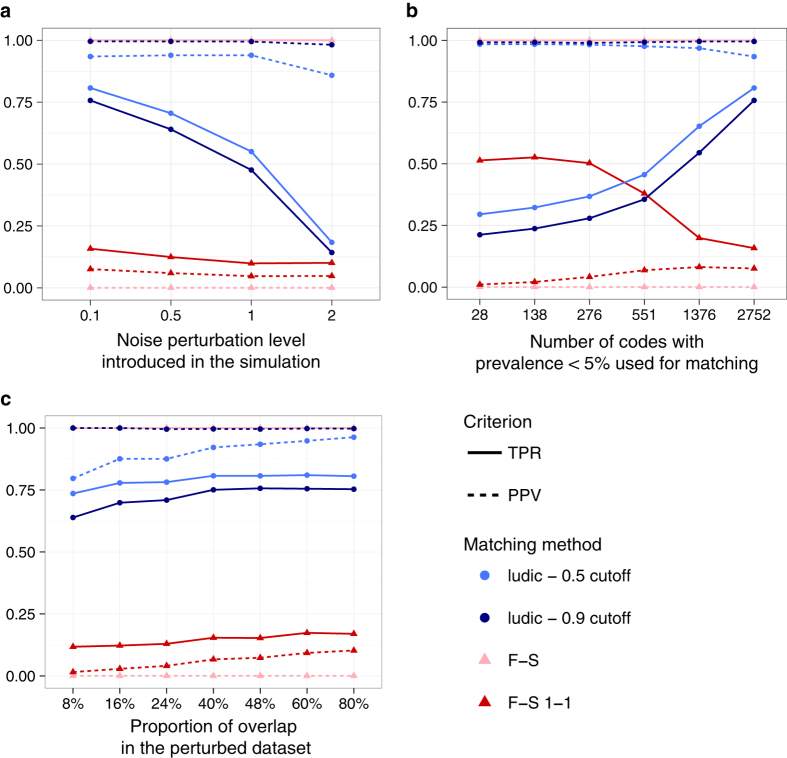
Matching accuracy on simulated data under various settings. (**a**) Impact of the discordance between the two datasets. (**b**) impact of rare codes. (**c**) impact of the proportion of overlapping patient between the two datasets. The figure shows the performance of the matching according to various simulation scenarios in terms of True Positive Rate (TPR) and Positive Predictive Value (PPV). F-S refers to the Fellegi-Sunter method while ludic denotes our proposed Bayesian approach.

Figure 1 displays both the Positive Predictive Value (PPV) and the True Positive Rate (TPR) under various simulation settings. As expected, the 0.9 cutoff has higher PPV, while the 0.5 cutoff has higher TPR under every circumstance. For both cutoffs, our method exhibits good PPV for most settings and a TPR > 0.5 when (a) the noise perturbation level is less than 1.0 and (b) at least 1000 codes with low prevalence were used for matching (Fig. 1a,b). On the contrary, the Fellegi-Sunter approach, even after the 1-1 matching modification, performs much worse with a PPV always below 20% and a TPR not much higher. This poor performance of the Fellegi-Sunter method is partly due to the fact that it does not distinguish between a (0, 0) concordance and a (1, 1) concordance, resulting in a high matching score for pairs with a high number of matching *0*’s. In addition, the Fellegi-Sunter method also does not account for discrepancies between the data sources. From Fig. 1b we also see that the more codes that are included in the matching procedure, the better the performance. In addition, although performance is acceptable with only a small overlap proportion between the two datasets, there is a noticeable improvement of the matching as the overlap proportion increases (Fig. 1c).

**Figure 2:**
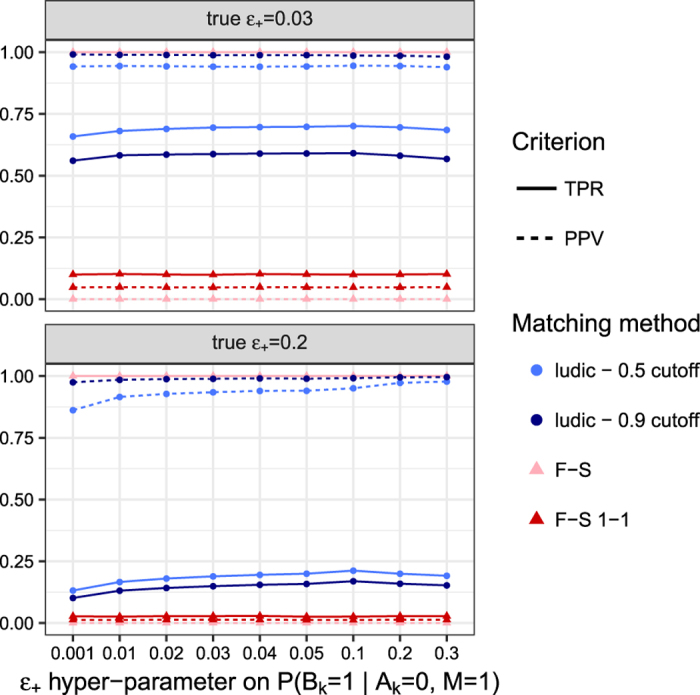
Matching accuracy according to ***ε***_+_. The figure shows the performance of the matching according to various value of the hyper-parameter *ε*_+_ which models the probability of discrepancy between the two datasets. The top panel displays results when true value of *ε*_+_ is 3%, while the bottom panel is for a true value of 20%.

In our simulations, we observed that the matching results are quite insensitive to the user input value for the hyper-parameter *ε*_+_ (representing the vector of marginal discrepancy rates between records, see Methods) provided that the simulated datasets shared enough diagnosis information, as shown in Fig. 2. A larger value of *ε*_+_ can slightly improve the performance if the true discrepancy is high, although a small fixed value of *ε*_+_ tends to work well. Concerning *ε*_−_ we have seen that it is generally better to provide a low value — this is expected in a context where diagnoses have low prevalence, making it unlikely to observe a discrepancy for a diagnosis given that it has been recorded in one of the datasets.

Note that our linkage algorithm requires that the two datasets contain overlapping diagnosis information (otherwise linkage is not feasible). For this reason, our approach will likely fail to link most de-identified datasets (which is the point of de-identifying a dataset). Yet it should be able to succeed in specific situations where diagnosis information overlap is sufficient (e.g., linking EHR data and claims data, or linking data collected over overlapping time span). In the next section, we apply our linkage algorithm to two sets of de-identified datasets on patients with rheumatoid arthritis (RA) with some matching patients, containing diagnosis codes recorded over overlapping time periods.

### Real world use-cases

We first applied our linkage algorithm to two de-identified datasets containing electronic medical records (EMR) from Partners HealthCare (a large hospital and physician network) in Boston, Massachusetts. Both EMR datasets were generated as part of prior research studies to look at patients with RA. The RA1 dataset contained 26,681 patients who had at least one ICD9 code of RA or had been tested for anti-cyclic citrullinated peptide. This dataset contained diagnosis codes recorded between 1/1/2002 and 12/31/2007, and these 26,681 patients together had 7,868 different unique diagnosis codes. The RA2 dataset contained 6,394 patients identified as having RA according to a phenotyping algorithm^24,25^, and it contained codes recorded between 1/1/2002 and 12/31/2012 (note the same start date but a later end date in RA2). The two datasets likely have overlapping patients, and we wanted to know if we can link the data from these two studies.

**Figure 3:**
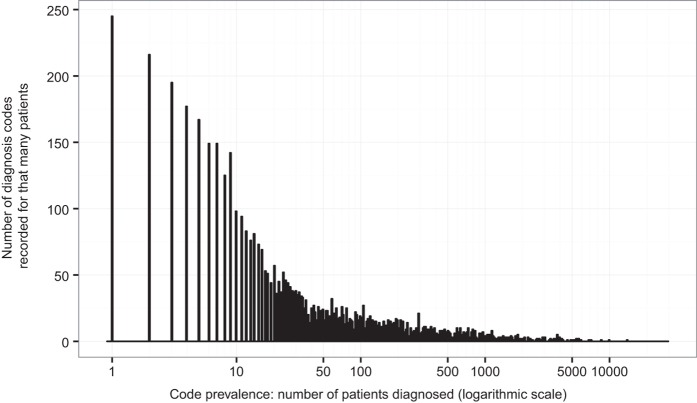
Histogram of diagnosis code prevalence in the RA datasets.

The 26,681 patients in the RA1 dataset together had 7,868 unique diagnosis codes from 2002 through 2007. Over the same time span as the RA1 dataset (2002 through 2007), 5,707 of the 6,394 patients in the RA2 dataset had at least one diagnosis recorded. These patients have 4,981 unique diagnosis codes through 2007, of which 4,936 also appear in the RA1 dataset. All patients in the RA2 dataset had at least one diagnosis when considering the full date range, from 2002 through 2012, with 6,086 unique diagnosis codes, of which 5,593 appear in the RA1 dataset. Patients in RA1 have a mean of 30.2 unique diagnosis codes; while patients in RA2 have a mean of 29.0 unique diagnosis codes recorded between 2002 and 2007 and 44.2 between 2002 and 2012. Table 1 summarizes these characteristics and Fig. 3 displays the histogram of diagnosis codes prevalence.

Ideally, the performance of the algorithm would be evaluated compared to a gold standard where the match between subjects in RA1 and RA2 are known. However, the true matching status for subjects in the two cohorts was not known. Fortunately, the full RA1 and RA2 datasets have laboratory test results and dates, which we used to create a silver standard as a proxy for true matching status in order to benchmark our method (the full RA1 and RA2 datasets lack demographic information, but they are not strictly de-identified since they contain dates). Subjects in RA1 with the same laboratory test results and dates in the RA2 dataset were assumed to be a true match, because it would be very unlikely that two different patients would have the exact same test result at the exact same date. This silver standard identified 3,831 subjects who are likely the same person in RA1 and RA2 according to this silver standard. Since not all patients have laboratory test results and the databases were created during different years, the silver standard is likely missing some true matches. Nevertheless, these silver standard labels should be sufficiently accurate to form a basis for evaluating the performance of our algorithm compared to the Fellegi-Sunter approach. We then removed the laboratory test results and dates from RA1 and RA2 to generate truly de-identified datasets where only diagnosis codes are available for linkage.

The two datasets show very few discrepancies (i.e., when for a true match a diagnosis is recorded in one database and not in the other) when only 6 years of records are considered, and exhibit similar average statistics in Table 1. This is expected, because most patients in the RA2 set are expected to be a subset of the RA1 set, although we still anticipate some discrepancy between the two datasets (even with the same 6-year time span) since the two datasets were frozen at different times and records got updated over time due to administrative reasons. When considering the whole 11 years of records available, the extra 5 years allowed the second dataset to record additional diagnosis codes not present in the first one, thus introducing more discrepancies between the two. Such discrepancies can be seen in the average number of diagnoses per patient among the silver standard matches, for instance (see Table 1).

Our method exhibits very good performance with both TPR and PPV always above 80%, even when considering the 11-year time span, as can be seen from Table 2. Due to computational limitations of the F-S method, we had to implement a blocking strategy using age to compute the F-S results. As in the numerical study, the Fellegi-Sunter method shows extremely poor performance without the 1-1 matching enforcement modification. And even with it, the PPV does not exceed 26%, even dropping to a low of 17% when considering the 11-year time span.

Similarly, and to further illustrate the relevance of our proposed method, we have also linked a subsample of the RA1 dataset with data from the BRASS registry cohort^26^. The RA1 dataset contains electronic medical record data, collected as part of routine care, as well as a Genetic Risk Score (GRS) for RA on 1,454 subjects, calculated as part of a research study^27^. The BRASS cohort is a prospective observational study that started in 2003 to enroll patients diagnosed with RA at the Robert Breck Brigham Arthritis Clinic in Boston, MA (USA). Notably it recorded longitudinal measurements of RA Disease Activity Score (DAS) for 1,130 patients, a composite score consisting of patient and physician reported information. DAS, a validated measure of RA disease activity used in clinical trials, is not routinely measured as part of clinical care. Linking those two sub-cohorts would enable studying the association between this GRS and the DAS in RA patients, an analysis that could not be performed in either of the datasets alone. There were 4,126 diagnosis codes recorded at least once in either database between 2002 and 2008 that were used for the linkage. Setting both *ε*_+_ and *ε*_+-_ to 0.01, and with a threshold at 0.5 (respectively 0.9) on the matching posterior probability, this linkage exhibited a PPV of 0.92 (resp. 0.98) and a TPR of 0.76 (resp. 0.71). Here, performance indicators were again computed against 442 silver standard matches as a proxy (created from date-time stamps on the diagnosis and lab codes).

## Discussion

In this work, we developed a method that can effectively use diagnosis codes to link two de-identified research datasets. Our approach presents three innovations compared to available state-of-the-art probabilistic linkage algorithms: i) our method uses only diagnosis codes, and no unique or direct identifiers are required; ii) our method takes into account the expected discrepancies across datasets in modeling; iii) our method does not need any training subsample for tuning, nor does it need any human intervention. In addition, our method is scalable to the use of thousands of diagnosis codes at once. Traditional approaches for probabilistic record linkage, based on Fellegi-Sunter seminal method were designed for using only a few informative PHI variables to identify matching patients across datasets. However, such methods are not equipped to deal with the high dimensionality of diagnosis codes and their sparse information content (see Supplementary Information for details).

Our method takes advantage of the likelihood framework to give more weight either to the presence or absence of a diagnosis. This is particularly useful for diagnosis codes as the presence of a diagnosis is usually much more informative than its absence. However, in case a particular code would be quite common and its absence would actually be more informative, the use of empirical Bayes priors for the marginal prevalence of codes takes this into account and adaptively tunes those weights according to the actual prevalence of each code in the data. Thus, our method naturally accounts for each diagnosis frequency. Because extremely rare codes might dominate other information too much, it can be useful to use a pre-filtering to discard such extremely rare codes. In addition, our method uses the constraint that a given patient record should be matched at most once, leveraging the information from all the available patient records when estimating the posterior probability of a match for a given pair.

In both our simulation study and our real use case, the Fellegi-Sunter method performs quite poorly. This can be explained because it was not designed to deal with either high-dimensional or sparse-binary variables, but rather with a small number of highly informative PHI variables. One way to improve the results from Fellegi-Sunter could be to compute agreement between two records differently, for instance treating a concurring diagnosis absence as non-informative, even though this could turn out to be harmful for highly prevalent diagnoses. On the contrary, our method tackles this issue by automatically tuning itself to the prevalence of each diagnosis.

Record linkage always requires some overlapping of information among the true matches between the datasets. If two datasets contain completely different information for the true matches, linkage will fail — regardless of the method used. Concerning diagnosis codes, there could be various reasons why information across databases would not overlap, such as disjoint time-periods for the record collection, or records from different healthcare systems. With less overlapping information, matches are all the less likely to be recovered by our approach. For instance, in the extreme case of no overlapping years, a few true matches might still be recovered for patients who have rare chronic conditions, but the sensitivity of the linkage would be quite poor. In practice, such degradation of shared information, across time or space, would give mediocre linkage performance indicated by low posterior matching probabilities.

EHRs can sometimes contain duplicate patients’ records. In this work, we assume that each record is unique in one dataset, which we take advantage of by enforcing 1-1 matching. In practical cases, one might be required to perform a deduplication step on each dataset before linking them. Deduplication itself can be viewed as a matching problem and our approach can be easily adapted to solve it, for instance by linking a dataset to itself. Another straightforward extension of the proposed method is the inclusion of demographic blocking if available and needed. This can be done by adding a likelihood term comparing those demographics and assigning a very high prior weight for disagreement. Also, one limitation of the method proposed here is that it uses only binarized diagnosis codes. It ignores the actual number of times clinicians have assigned the same diagnosis code to a patient through multiple visits. A natural extension of our method would be to derive the likelihood for actual count-data instead of binary distributed codes, in order to use more information. This could be done for instance by using bivariate zero-inflated distributions^28^ in the likelihood. A final limitation is the potential risk to patients from re-identifying a de-identified dataset by linking it to a dataset containing PHI. Often the data use agreements for research datasets forbid any attempts at doing record linkage for this reason, but the use of formal privacy models could help mitigate that risk^29,30^.

Although we expect the proposed patient linkage algorithm for de-identified research data using diagnosis codes to be broadly applicable, the good performance of the algorithm in real applications thus far has only been demonstrated in linking RA cohorts. Additional empirical assessment of its linkage performance in a broader set of real applications is warranted to ascertain its general applicability.

Various factors can influence the linkage performance, such as the record time-periods, the record health care systems, the prevalence of diagnoses, quality and accuracy of the records, health care utilization, or the proportion of patient overlap. However, based on both realistic simulations and two real world use-cases, we show that it is possible to achieve good linkage performance using only diagnosis codes as long as i) there is sufficient diagnosis information shared between the two datasets, and ii) there are enough rare diagnoses. We provide an open-source implementation of the proposed method, with complete documentation, in the R package *ludic* (Linkage Using DIagnosis Codes) available on CRAN. In addition, the *ludic* package also includes two anonymized diagnosis code datasets from our real use-case (along with a silver standard of true matches) for benchmarking future record linkage methods.

## Methods

### Data sources

We used three data sources for this study. The first was a de-identified administrative claims dataset from a U.S. based nationwide health insurer, which included all billing diagnosis codes recorded in 2010 for women between 50 and 69 years old with zip codes 021XX. Procedure codes are not included here, but their inclusion would be straightforward in the proposed approach. This dataset provided us a basis for generating diagnosis codes with realistic frequency distributions in our simulation studies. The second data source is from the EHR of patients with diagnostic codes of rheumatoid arthritis (RA) at Partners Healthcare in Boston, Massachusetts. The third data source is from the BRASS RA registry cohort which enrolls patients diagnosed with RA at the Robert Breck Brigham Arthritis Clinic in Boston, MA (USA).

We used the second and third data sources for validating the performance of our algorithm in real world settings. We first tested the algorithm’s performance using two different de-identified EHR datasets of patients with diagnostic codes of RA. The two datasets contain overlapping but not identical subjects with RA identified via an EHR phenotyping algorithm^24,25^ in 2009 for the first dataset (RA1), and then again in 2014 for the second dataset (RA2). For each cohort, the structured data, including all diagnosis codes, were previously extracted from the EHR and stored as a de-identified dataset. The original datasets were actually limited datasets, containing not only diagnoses, but also the exact dates and results of laboratory tests. We first used these additional variables to deterministically link the datasets, based on the assumption that the dates and test results would be unique enough in these relatively small cohorts to perform accurate deterministic linkage. Because we cannot guarantee that this linkage is perfectly correct, we call this a “silver standard” rather than gold standard mapping. We then removed the dates and test results to create two de-identified RA datasets, which we could use to test our probabilistic record linkage algorithm using only diagnosis codes and compare our results to this silver standard mapping. We further validated our algorithm by linking the RA1 dataset with the BRASS cohort which contains longitudinal disease activity scores, inflammation markers as well as EHR data on diagnostic codes.

The RA studies are examples of real-life use cases demonstrating the need for a probabilistic algorithm. The RA1 cohort contains structured EHR data, single nucleotide polymorphism (SNP) data, auto-antibody marker measurements, as well as abstracted information from clinical notes obtained using chart review. In the RA2 cohort, additional data were abstracted from clinical notes, and biomarkers as well as genetic methylation levels were measured. While the genetic and biomarker data are non-overlapping between the two cohorts, there is partial overlap for the structured EHR data, namely diagnosis codes among subjects in both cohorts. Being able to link the datasets would enable combined analysis of the two complementary sets of genetic and biomarker data between the two cohorts. Linking RA1 with the BRASS cohort would enable association studies on the effect of auto-antibody biomarkers on the longitudinal disease activity or inflammation markers in RA patients.

The Institutional Review Board at Harvard University approved the use of the administrative claims data. The Institutional Review Board at Partner’s Healthcare Systems approved the use of the two RA datasets for probabilistic record linkage.

### Linkage algorithm

**Figure 4:**
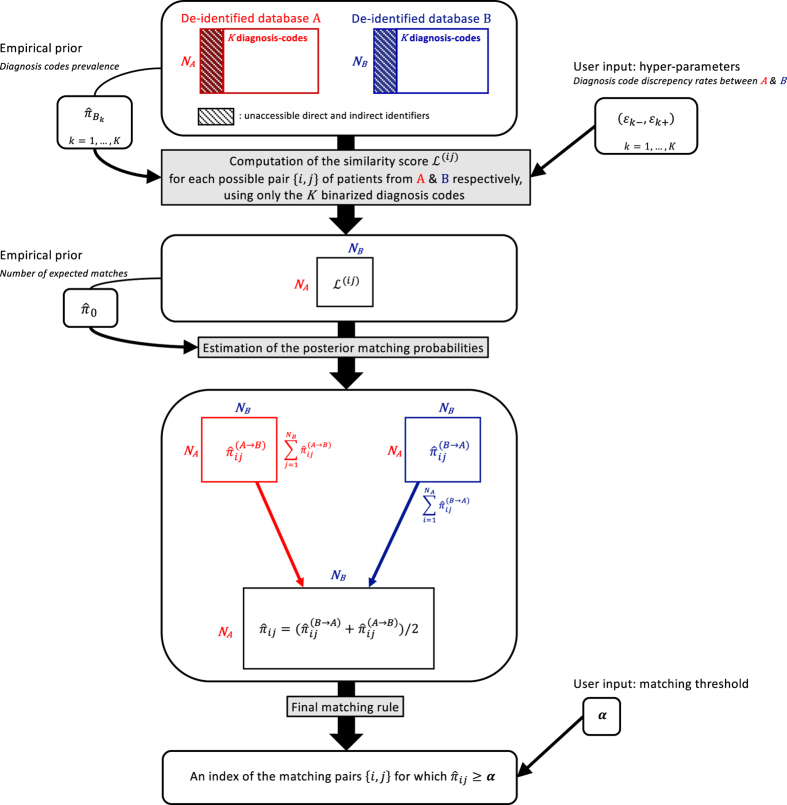
Workflow of the linkage algorithm.

Our goal is to link dataset **A** consisting of *n*_*A*_ records to dataset **B** consisting of *n*_*B*_ records. Assume that *K* common features, such as diagnosis codes, are recorded in both datasets. For *i*=1, …, *n*_*A*_ and *j*=1, …, *n*_*B*_, let *M*_*ij*_ be the binary indicator of whether these two records are a true match, $A^{\left(i\right)}=(A_{1}^{\left(i\right)},\ldots ,A_{K}^{\left(i\right)})$ represent the *K* features for patient *i* from **A**, and $B^{\left(j\right)}=(B_{1}^{\left(j\right)},\ldots ,B_{K}^{\left(j\right)})$ represent the *K* features for patient *j* from **B**. All K features are assumed to be binary, e.g., presence or absence of a diagnosis code. A key component in our linkage algorithm is the posterior probability of being a match for each possible pair {*i*, *j*} of patients from **A** and **B**: $\left\{\pi_{ij}=\mathbb{P}\left(M_{ij}=1|A,B\right):i=1,\ldots ,n_{A},j=1,\ldots ,n_{B}\right\}$. We next detail key components of the algorithm. See Fig. 4 for the complete workflow of our algorithm.

### Bayesian model

To compute the posterior matching probability *π*_*ij*_, we first compute a similarity measure for **A**^(*i*)^ and **B**^(*j*)^ through a likelihood formulation. To this end, we assume that the respective distributions of **A**^(*i*)^ and **B**^(*j*)^ do not depend on whether they can be matched with $\pi_{A_{k}}=P(A_{k}^{\left(i\right)}=1)$ and $\pi_{B_{k}}=P(B_{k}^{\left(j\right)}=1)$. To allow discrepancy between the records in the two datasets, let $\varepsilon_{k-}=\mathbb{P}(B_{k}^{\left(j\right)}=0|A_{k}^{\left(i\right)}=1,M_{ij}=1)$ and $\varepsilon_{k+}=\mathbb{P}(B_{k}^{\left(j\right)}=1|A_{k}^{\left(i\right)}=0,M_{ij}=1)$ as the respective marginal discrepancy rates for the *k*^*th*^ feature between records. These hyper-parameters can be related to the *m*_*k*_ and *u*_*k*_ parameters from the Fellegi-Sunter approach by the following equations: $m_{k}=\pi_{A_{k}}\left(1-\varepsilon_{k-}\right)+(1-\pi_{A_{k}})(1-\varepsilon_{k+})$ and $u_{k}=\pi_{A_{k}}\pi_{B_{k}}+(1-\pi_{A_{k}})(1-\pi_{B_{k}})$. The log-likelihood ratio for observing $A_{k}^{\left(i\right)}$and $B_{k}^{\left(j\right)}$is ${ℒ}_{k}\left(A_{k}^{\left(i\right)},B_{k}^{\left(j\right)}\right)$, where

$$\begin{array}{c} {ℒ}_{k}\left(a,b\right)=\log\left(\frac{\mathbb{P}(A_{k}^{\left(i\right)}=a,B_{k}^{\left(j\right)}=b|M^{\left(ij\right)}=1)}{\mathbb{P}(A_{k}^{\left(i\right)}=a,B_{k}^{\left(j\right)}=b|M^{\left(ij\right)}=0)}\right)=1_{a=1,b=1}\log\left(\frac{{\bar{\varepsilon }}_{k-}}{\pi_{B_{k}}}\right)\\ +1_{a=0,b=0}\log\left(\frac{{\bar{\varepsilon }}_{k+}}{{\bar{\pi }}_{B_{k}}}\right)+1_{a=1,b=0}\log\left(\frac{\varepsilon_{k-}}{{\bar{\pi }}_{B_{k}}}\right)+1_{a=0,b=1}\log\left(\frac{\varepsilon_{k+}}{\pi_{B_{k}}}\right) \end{array}$$

where for brevity we write $\bar{\varepsilon }=1-\varepsilon$ for any *ε*. Then we define the similarity measure between **A**^(*i*)^ and **B**^(*j*)^ as ${ℒ}^{\left(ij\right)}={\sum^{\;}}_{k=1}^{K}{ℒ}_{k}\left(A_{k}^{\left(i\right)},B_{k}^{\left(j\right)}\right)$ which essentially is the log-likelihood under a naive Bayes assumption, that is commonly used in the context of record linkage^11,15,18^ and that maintains good classification performance even when the independence assumption does not hold in practice^31–33^.

To calculate the posterior probability *π*_*ij*_, we further impose an assumption that the probabilities of a record in **A**, **A**^(*i*)^, being matched to either one record in **B** or none sum up to 1. This essentially would assign this probability to 0.5 if **A**^(*i*)^ is matched to two identical records in **B**. For patient *i* from **A**, we define the random variable $X_{i}\in \{0,1,2,\ldots ,j,\ldots ,N_{B}\}$ indexing the patient in **B** who is a match, with *X*_*i*_=0 indicating that no match is found. The posterior probability of *X*_*i*_=*j* can be shown to be (see Supplementary Information for details):

$$\pi_{ij}^{\left(A\to B\right)}=\mathbb{P}(X_{i}=j|A^{\left(i\right)},B)=\frac{\exp\left({ℒ}^{\left(ij\right)}+\mathrm{logit}\left(\pi_{0}\right)\right)}{1+\sum_{\ell =1}^{N_{B}}\exp\left({ℒ}^{\left(i\ell \right)}+\mathrm{logit}(\pi_{0})\right)}$$

where ***π***_0_ is the prior probability of matching for a random pair from **A** and **B**. Similarly, we let the random variable *Y*_*j*_ be the matching index of patient *j* from **B** in **A** and its posterior probability given the data is:

$$\pi_{ij}^{\left(B\to A\right)}=\mathbb{P}(Y_{j}=i|A,B^{\left(j\right)})=\frac{\exp\left({ℒ}^{\left(ij\right)}+\mathrm{logit}\left(\pi_{0}\right)\right)}{1+\sum_{\ell =1}^{N_{A}}\exp({ℒ}^{\left(\ell j\right)}+\mathrm{logit}(\pi_{0}))}$$

### Hyper-parameters

The posterior probabilities involve several unknown hyper-parameters including *π*_*Bk*_, *ε*_*k*-_ and *ε*_*k*+_ for each *k* in {1, …, *K*} and *π*_0_. It is straightforward to estimate *π*_*Bk*_ empirically using sample fractions in the data directly, as this represents the prevalence of the feature *k* in the dataset **B**. To estimate the prior matching probability *π*_0_, we note that the observed similarity measures $\vec{ℒ}=\{{ℒ}^{\left(ij\right)},i=1,\ldots ,n_{A},j=1,\ldots ,n_{B}\}$ follow a mixture distribution with density *π*_0_*g* + (1 − *π*_0_)*f*, where *g* and *f* are the respective density functions of ${ℒ}^{\left(ij\right)}|M_{ij}=1$ and ${ℒ}^{\left(ij\right)}|M_{ij}=0$. Since a vast majority of the pairs are not matches and the similarity measures among matched pairs are expected to follow a distribution very different from *f*, we estimate *π*_0_, as the fraction of pairs with ${ℒ}^{\left(ij\right)}$ exceeding a threshold value ${\hat{c}}_{0}$ chosen as the furthest inflexion point of *f* on the right with *f* estimated by fitting a skew-t distribution to $\vec{ℒ}$. See Supplementary Information for details. Finally, the discrepancy rates $\left\{\left(\varepsilon_{k-},\varepsilon_{k+}\right),k=1,\ldots ,K\right\}$ are generally not directly estimable in datasets without gold standard labels on which pairs are matched. Again, they can instead be roughly estimated from the data using only pairs with high ${ℒ}^{\left(ij\right)}$ (for instance those exceeding ${\hat{c}}_{0}$), or one can provide an educated guess according to prior knowledge.

### Final matching rule

For the pair **A**^(*i*)^ and **B**^(*j*)^, we estimate the posterior probability of being a match as ${\hat{\pi }}_{ij}=${${\hat{\pi }}_{ij}^{\left(B\to A\right)}+{\hat{\pi }}_{ij}^{\left(A\to B\right)}\}/2$, where ${\hat{\pi }}_{ij}^{\left(B\to A\right)}$ and ${\hat{\pi }}_{ij}^{\left(A\to B\right)}$ are the respective estimates of $\pi_{ij}^{\left(B\to A\right)}$ and $\pi_{ij}^{\left(A\to B\right)}$ obtained by plugging in the estimated hyper-parameters. Finally, a pair is identified as a match if ${\hat{\pi }}_{ij}\geq \alpha$, where the threshold *α* requires user input and its choice depends on the goal of the study. A straightforward sensible cutoff value is 0.5 (more chances of the pair being a match than not), while a more stringent threshold of 0.9 can be used if the tolerance for false matches in the study is very low.

### Code availability

Open-source software implementing the proposed method is available together with complete documentation in the R package *ludic* (Linkage Using DIagnosis Codes) from the Comprehensive R Archive Network (CRAN) at https://CRAN.R-project.org/package=ludic.

### Anonymized data usage notes

The R package *ludic* also includes an anonymized version of the binarized diagnosis code data from the RA1 and RA2 datasets, for both 6-year and 11-year time span. In accordance with the IRB, the ICD-9 diagnosis codes have also been masked and randomly reordered, replaced by meaningless names. Finally, the silver standard matching pairs are also provided to allow the benchmarking of methods for probabilistic record linkage using diagnosis codes.

### Data Availability

The “RA1” and “RA2” data that support the findings of this study are available from the Comprehensive R Archive Network (CRAN) as part of the R package *ludic*, at https://CRAN.R-project.org/package=ludic. See the Anonymized data usage notes for more information.

## Additional information

**How to cite this article**: Hejblum, B. P. *et al*. Probabilistic record linkage of de-identified research datasets with discrepancies using diagnosis codes. *Sci. Data*. 6:180298 doi: 10.1038/sdata.2018.298 (2019).

**Publisher’s note**: Springer Nature remains neutral with regard to jurisdictional claims in published maps and institutional affiliations.

## Supplementary Material

Supplementary Information

## Figures and Tables

**Table 1:** Characteristics of the real datasets.

Dataset	Time span^a^	Number of patients with at least 1 diagnosis code	Number of diagnosis code recorded at least once	Average number of diagnoses per patient	Average number of diagnoses per patient among silver standard matches
RA1	6 years	26,681	7,868	30.2	33.6
RA2	6 years	5,707	4,981	29.0	33.3
RA2	11 years	6,394	6,086	44.2	54.0
^a^The 6-year time span includes codes from 1/1/2002 through 12/31/2007, while the 11-year time span includes codes from 1/1/2002 through 12/31/2012.					

**Table 2:** Performance matching 2 real use case datasets.

Data time span	Matching method	Number of matches	TPR^a^	PPV^a^	Computing time
6 years	0.5 cutoff	4,369	0.93	0.81	96 s^b^
6 years	0.9 cutoff	4,179	0.91	0.84	96 s^b^
6 years	F-S blocked	2,594,443	0.81	<0.01	49 min^b^
6 years	F-S blocked 1-1	5,696	0.38	0.26	49 min^b^
6 years	F-S	—	—	—	> 4 days^c^
6 years	F-S 1-1	—	—	—	> 4 days^c^
11 years	0.5 cutoff	4,043	0.84	0.80	96 s^b^
11 years	0.9 cutoff	3,625	0.80	0.84	96 s^b^
11 years	F-S blocked	2,898,367	0.80	<0.01	62 min^b^
11 years	F-S blocked 1-1	6,356	0.29	0.17	62 min^b^
11 years	F-S	—	—	—	> 4 days^c^
11 years	F-S 1-1	—	—	—	> 4 days^c^
^a^Based on the 3,831 silver standard true matches.					
^b^Using a 3.5 GHz Intel Core i7 processor with 32 GB of memory available.					
^c^Using a 3.6 GHz Intel Xeon 5600 series processor with 96 GB of memory available.					
