# Supplementary material for: Probabilistic record linkage of de-identified research datasets with discrepancies using diagnosis codes
Source: Sci Data. 2019 Jan 8;6:180298. doi: 10.1038/sdata.2018.298 (PMC6326114; doi:10.1038/sdata.2018.298)
Supplement: Supplementary Information [file sdata2018298-s1.pdf]

Probabilistic Record Linkage of De-Identified Research Datasets with Discrepancies Using Diagnosis Codes — Supplementary Information

Boris P. Hejblum[1,2*], Griffin M. Weber[3], Katherine P. Liao[4], Nathan P. Palmer[3], Susanne Churchill[3], Nancy A. Shadick[4], Peter Szolovits[5], Shawn N. Murphy[6,7], Isaac S. Kohane[3], Tianxi Cai[1,3]

[1]Department of Biostatistics, Harvard T.H. Chan School of Public Health, Boston, MA, USA;
[2]Univ. Bordeaux, ISPED, Inserm Bordeaux Population Health Research Center, UMR 1219, Inria SISTM, Bordeaux F-33000, France;
[3]Department of Biomedical Informatics, Harvard Medical School, Boston, MA, USA;
[4]Division of Rheumatology, Immunology, and Allergy, Brigham and Women's Hospital, Boston, MA, USA;
[5]Computer Science and Artificial Intelligence Laboratory (CSAIL), Massachusetts Institute of Technology, Cambridge, MA, USA;
[6]Department of Neurology, Massachusetts General Hospital, Boston, MA, USA
[7]Research IS and Computing, Partners HealthCare, Charlestown, MA, USA

Contents

A Posterior probabilities calculation details 2

B Fitting a skew-t distribution to observed $\mathcal{L}$ 3

C EHR data from Partners RA patients 4

D On the failure of the Fellegi-Sunter method for matching using diagnosis codes 4

E EHR data for the BRASS cohort patients 5

*bhejblum@hsph.harvard.edu

A Posterior probabilities calculation details

Applying the Bayes theorem to the log-ratio, one finds:

$$\log\left(\frac{\mathbb{P}(M^{(ij)}=1 \mid \boldsymbol{A}^{(i)}=\boldsymbol{a}, \boldsymbol{B}^{(j)}=\boldsymbol{b})}{\mathbb{P}(M^{(ij)}=0 \mid \boldsymbol{A}^{(i)}=\boldsymbol{a}, \boldsymbol{B}^{(j)}=\boldsymbol{b})}\right) = \mathcal{L}^{(ij)} + \text{logit}(\pi_0) \tag{1}$$

where $\mathcal{L}^{(ij)} = \sum_{k=1}^{K} \mathcal{L}_k^{(ij)}$. It then follows that:

$$\begin{aligned}
\log\left(\frac{\mathbb{P}(\mathcal{X}_i = j \mid A^{(i)}, B^{(j')}, j'=1,2,\ldots,N_B)}{\mathbb{P}\left(\mathcal{X}_i = 0 \mid A^{(i)}, B^{(j')}, j'=1,2,\ldots,N_B\right)}\right) &= \log\left(\frac{\mathbb{P}(M^{(ij)}=1 \mid A^{(i)}, B^{(j)})}{\mathbb{P}(M^{(ij)}=0 \mid A^{(i)}, B^{(j)})}\right) \qquad (2)\\
&\quad + \log\left(\frac{\prod_{j'\neq j} \mathbb{P}(M^{(ij')}=0 \mid A^{(i)}, B^{(j')})}{\prod_{j'\neq j} \mathbb{P}(M^{(ij')}=0 \mid A^{(i)}, B^{(j')})}\right)\\
&= \mathcal{L}^{(ij)} + \text{logit}(\mathbb{P}(M=1))
\end{aligned}$$

Note that:

$$\begin{aligned}
\frac{1}{\mathbb{P}(\mathcal{X}_i = 0 \mid A^{(i)}, B^{(j')}, j'=1,2,\ldots,N_B)} &= \frac{\sum_{\ell=0}^{N_B} \mathbb{P}(\mathcal{X}_i = \ell \mid A^{(i)}, B^{(j')}, j'=1,2,\ldots,N_B)}{\mathbb{P}(\mathcal{X}_i = 0 \mid A^{(i)}, B^{(j')}, j'=1,2,\ldots,N_B)} \qquad (3)\\
&= 1 + \sum_{\ell=1}^{N_B} \frac{\mathbb{P}(\mathcal{X}_i = \ell \mid A^{(i)}, B^{(j')}, j'=1,2,\ldots,N_B)}{\mathbb{P}(\mathcal{X}_i = 0 \mid A^{(i)}, B^{(j')}, j'=1,2,\ldots,N_B)}\\
&= 1 + \sum_{\ell=1}^{N_B} \exp\left(\mathcal{L}^{(i\ell)} + \text{logit}(\pi_0)\right)
\end{aligned}$$

B Fitting a skew-t distribution to observed $\mathcal{L}$

There are several way to parametrize skew t distribution. We use the following:

$$f_{\mathcal{ST}}(x,m,s,\nu,\xi) = \frac{2\sigma}{s(\xi+1/\xi)}\sqrt{\frac{\nu}{\nu-2}}f_{\mathcal{T}}\left(\sqrt{\frac{\nu}{\nu-2}}\,\frac{1}{\xi}\left(\frac{x-m}{s}\sigma+\mu\right),\nu\right)\mathbb{1}_{\left\{\left(\frac{x-m}{s}\sigma+\mu\right)\geq 0\right\}} \tag{4}$$

$$\frac{2\sigma}{s(\xi+1/\xi)}\sqrt{\frac{\nu}{\nu-2}}f_{\mathcal{T}}\left(\sqrt{\frac{\nu}{\nu-2}}\,\xi\left(\frac{x-m}{s}\sigma+\mu\right),\nu\right)\mathbb{1}_{\left\{\left(\frac{x-m}{s}\sigma+\mu\right)<0\right\}} \tag{5}$$

where

- $\mu = m_1(\xi - 1/\xi)$
- $m_1 = \dfrac{2\sqrt{\nu-2}}{(\nu-1)\beta}$
- $\beta = \dfrac{\Gamma\left(\frac{1}{2}\right)\Gamma\left(\frac{\nu}{2}\right)}{\Gamma\left(\frac{\nu+1}{2}\right)}$
- $\sigma = \sqrt{(1-m_1^2)(\xi^2+1/\xi^2)+2m_1^2-1)}$
- $f_{\mathcal{T}}$ is the density function of a Student's t distribution:

$$f_{\mathcal{T}}(y,\nu) = \frac{\Gamma\left(\frac{\nu+1}{2}\right)}{\sqrt{\pi\nu}\,\Gamma\left(\frac{\nu}{2}\right)}\left(1+\frac{y^2}{\nu}\right)^{-\frac{\nu+1}{2}}$$

In order to estimate π_0, we compute the first and second x-derivative of $f_{\mathcal{ST}}$ to determine the inflexion point the further left of density curve. Therefore, we focus on the case where $\left(\frac{x-m}{s}\sigma+\mu\right)\geq 0$:

So $f_{\mathcal{ST}}(x,m,s,\nu,\xi) = c\left(1+\dfrac{1}{\xi^2(\nu-2)}\left(\dfrac{x-m}{s}\sigma+\mu\right)^2\right)^{-\frac{\nu+1}{2}}$

where c does is a constant for x. Then

$$\frac{\mathrm{d}}{\mathrm{d}x} f_{\mathcal{ST}}(x, m, s, \nu, \xi) = d\left(\frac{x-m}{s}\sigma + \mu\right)\left(1 + \frac{1}{\xi^2(\nu-2)}\left(\frac{x-m}{s}\sigma + \mu\right)^2\right)^{-\frac{\nu+3}{2}}$$

where $d = -c\,\frac{(\nu+1)\sigma}{\xi^2(\nu-2)s}$, and

$$\begin{aligned}\frac{\mathrm{d}^2}{\mathrm{d}x^2} f_{\mathcal{ST}}(x, m, s, \nu, \xi) =& d\frac{\sigma}{s}\left(1 + \frac{1}{\xi^2(\nu-2)}\left(\frac{x-m}{s}\sigma + \mu\right)^2\right)^{-\frac{\nu+5}{2}} \\ & \left[1 - \frac{\nu+2}{\xi^2(\nu-2)}\left(\frac{x-m}{s}\sigma + \mu\right)^2\right]\end{aligned}$$

Finally we use $\widehat{\pi_0} = \min\left\{x \;\middle|\; \frac{\mathrm{d}}{\mathrm{d}x} f_{\mathcal{ST}}(x, m, s, \nu, \xi) < \varepsilon \text{ and } \frac{\mathrm{d}^2}{\mathrm{d}x^2} f_{\mathcal{ST}}(x, m, s, \nu, \xi) < \varepsilon\right\}$

C EHR data from Partners RA patients

The dataset we used here has two more years of follow-up compared to the one described in Liao *et al.* [1]. The 2 additional years of follow-up allowed us to identify more patients when the algorithm used in Liao *et al.* was thus ran on a larger set of patients. Also, there was a change in the Electronic Health Record system that occurred in late 2001, so we only used records starting in 2002 onwards to ensure data coherence.

D On the failure of the Fellegi-Sunter method for matching using diagnosis codes

The Fellegi-Sunter method for record linkage [2] fails in the context of diagnosis codes because it does not differentiate agreement (between both datasets) for the presence or for the absence of a diagnosis code. However, agreement for the presence of a diagnosis code is often much more informative for

matching than agreement for its absence. Our proposed approach has the advantage of not only differentiating the agreement weights between presence and absence, but also to automatically tune them according to the prevalence of a diagnosis (considering rarer codes as more informative). For this reason, Fellegi-Sunter will fail when using diagnosis codes, even when reaching matching weight higher than 16.6 in the context presented of Figure 1 (the lower bound given by Cook *et al.* [3] approach supposed to ensure a successful linkage with a probability of selecting true matches above 95%).

E EHR data for the BRASS cohort patients

As described in Iannaccone *et al.* [4], BRASS patients were recruited prospectively through the clinic and data are collected through patient interviews and questionnaires. When BRASS patients are enrolled, they are assigned a BRASS study ID which is linked to their medical record number (MRN). For the study presented here, IRB approval was granted to extract the EHR data for the BRASS patients using their MRNs. Patients in the BRASS study have blood samples drawn and analyzed at a separate laboratory, that are not available in the EHR. The labs used to construct the silver standard in the study presented here are part of their routine follow-up in the Arthritis Center and are extracted from the EHR using the method described above.

References

1. Liao, K. P., Ananthakrishnan, A. N., Kumar, V., Xia, Z., Cagan, A., Gainer, V. S., Goryachev, S., Chen, P., Savova, G. K., Agniel, D., Churchill, S., Lee, J., Murphy, S. N., Plenge, R. M., Szolovits, P., Kohane, I., Shaw, S. Y., Karlson, E. W. & Cai, T. Methods to Develop an Electronic Medical Record Phenotype Algorithm to Compare the Risk of Coronary Artery Disease across 3 Chronic Disease Cohorts. *PLOS ONE* **10,** e0136651 (2015).

2. Fellegi, I. P. & Sunter, A. B. A Theory for Record Linkage. *Journal of the American Statistical Association* **64,** 1183–1210 (1969).

3. Cook, L. J., Olson, L. M. & Dean, J. M. Probabilistic Record Linkage: Relationships between File Sizes, Identifiers, and Match Weights. *Methods of Information in Medicine* **40,** 196–203 (2001).

4. Iannaccone, C. K., Lee, Y. C., Cui, J., Frits, M. L., Glass, R. J., Plenge, R. M., Solomon, D. H., Weinblatt, M. E. & Shadick, N. A. Using Genetic and Clinical Data to Understand Response to Disease-Modifying Anti-Rheumatic Drug Therapy: Data from the Brigham and Women's Hospital Rheumatoid Arthritis Sequential Study. *Rheumatology* **50,** 40–46 (2011).
